# Data supporting the structural and functional characterization of Thrombin‐Activatable Fibrinolysis Inhibitor in breast cancer

**DOI:** 10.1016/j.dib.2015.10.043

**Published:** 2015-11-14

**Authors:** Manal S. Fawzy, Eman A. Toraih

**Affiliations:** a Department of Medical Biochemistry, Faculty of Medicine, Suez Canal University, Ismailia, Egypt; b Department of Histology and Cell Biology (Genetics Unit), Faculty of Medicine, Suez Canal University, Ismailia, Egypt

## Abstract

The data in this paper is related to the research article entitled “Thrombin-activatable fibrinolysis inhibitor Thr325Ile polymorphism and plasma level in breast cancer: A pilot study” (Fawzy et al., 2015) [1]. Many emerging studies have begun to unravel the pathophysiologic role of the fibrinolytic system in breast cancer (BC) progression (Zorio et al., 2008) [2]. Activation of the fibrinolytic plasminogen/plasmin system results in degradation of protein barriers, thereby mediating cell migration essential for tumor growth, angiogenesis, and dissemination (Castellino and Ploplis, 2005) [3]. In the current study, *in silico* data analysis of Thrombin-Activatable Fibrinolysis Inhibitor (*TAFI*) gene and protein has been done. Data have been retrieved from several databases mentioned in details in the text. Determination and analysis of the structural and functional impact of TAFI and its expression could help elucidate the contribution of the TAFI pathway to acquired hemostatic dysfunction and will form the basis of potential therapeutic strategies to manipulate this pathway. An inhibition of TAFI (e.g. by FXI inhibitors) will offer the therapeutic possibilities to improve the decreased fibrinolysis and increase the efficiency of fibrinolytic therapy in thrombotic disorders including cancer.

**Specifications Table**

**Table:** 

Subject area	*Bioinformatics and Biology*
More specific subject area	*Molecular Biology*
Type of data	*Figure, text*
How data was acquired	*Databases research*
Data format	*Analyzed*
Experimental factors	*None*
Experimental features	*None*
Data source location	*Ensembl, GeneCards, UniProtKB, NextProt beta, Phobius predictions, PeptideAtlas, String, Compartment, Vega Genome Browser release 62, dbSNP release 142, MGI phenotype, PolyPhen v2.0, MutPred*
Data accessibility	*The data are supplied with this article*

**Value of the data**

• This data provides a comprehensive *in silico* analysis of the structural and functional characterization of TAFI and its expression.
• The data are useful for understanding the effect of TAFI variants on its structure and function.
• This data may provide insight for determining the role of future drug treatment to inhibit TAFI function especially in cancer cases.

## 1 Data, experimental design, materials and methods

### 1.1 TAFI activation and function

**Fig. 1:**
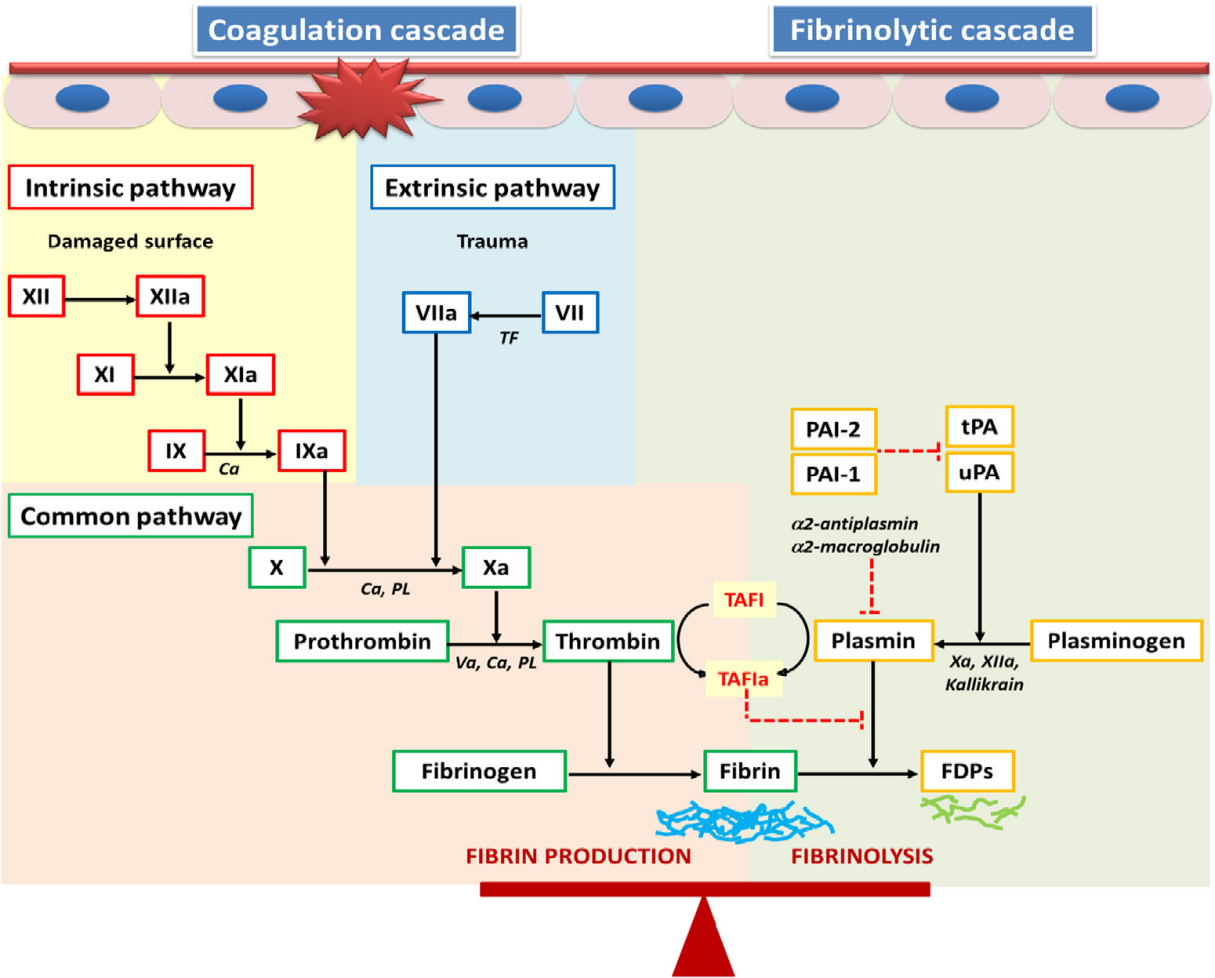
Coagulation and fibrinolytic cascades. Ca, calcium; FDPs, fibrin degradation product; PAI-1 and PAI-2; plasminogen activator inhibitors; PL, phospjolipids; TAFI, thrombin activatable fibrinolysis inhibitor; TF, tissue factor; tPA, tissue plasminogen activator; uPA, urokinase plasminogen activator. Both intrinsic and extrinsic pathways involved with a series of sequential cleavage events which ends with thrombin activation from its zymogen prothrombin. Active thrombin can then catalyze the polymerization of fibrin monomers which converts soluble fibrinogen into an insoluble fibrin matrix. As the clot forms, circulating red blood cells, white blood cells, and platelets become incorporated into its structure. In addition, fibrin becomes cross-linked providing further structural stability. On the other hand, fibrinolysis, through the action of plasmin, prevents unnecessary accumulation of intravascular fibrin and enables the removal of thrombi. Plasmin is generated from the zymogen plasminogen on the surface of the fibrin clot by either tissue plasminogen activator (tPA) or urokinase (uPA) [3]. Proteolysis of fibrin gives rise to soluble fibrin degradation products (FDPs), some of which have immunomodulatory and chemotactic functions [2]. The coagulation and fibrinolytic systems are highly regulated and interrelated through mechanisms that insure balanced hemostasis. The molecular linker between the two processes, TAFI, is first produced as a proenzyme that is activated by thrombin or plasmin generated during the coagulation cascade. The active form, TAFIa, inhibits fibrinolysis by cleaving off C-terminal lysine residues from partially degraded fibrin. These residues act as a template onto which both tPA and plasminogen bind thereby enhancing the catalytic efficiency of plasmin formation. Cleavage of these basic amino acids down-regulates fibrinolysis.

**Fig. 2:**
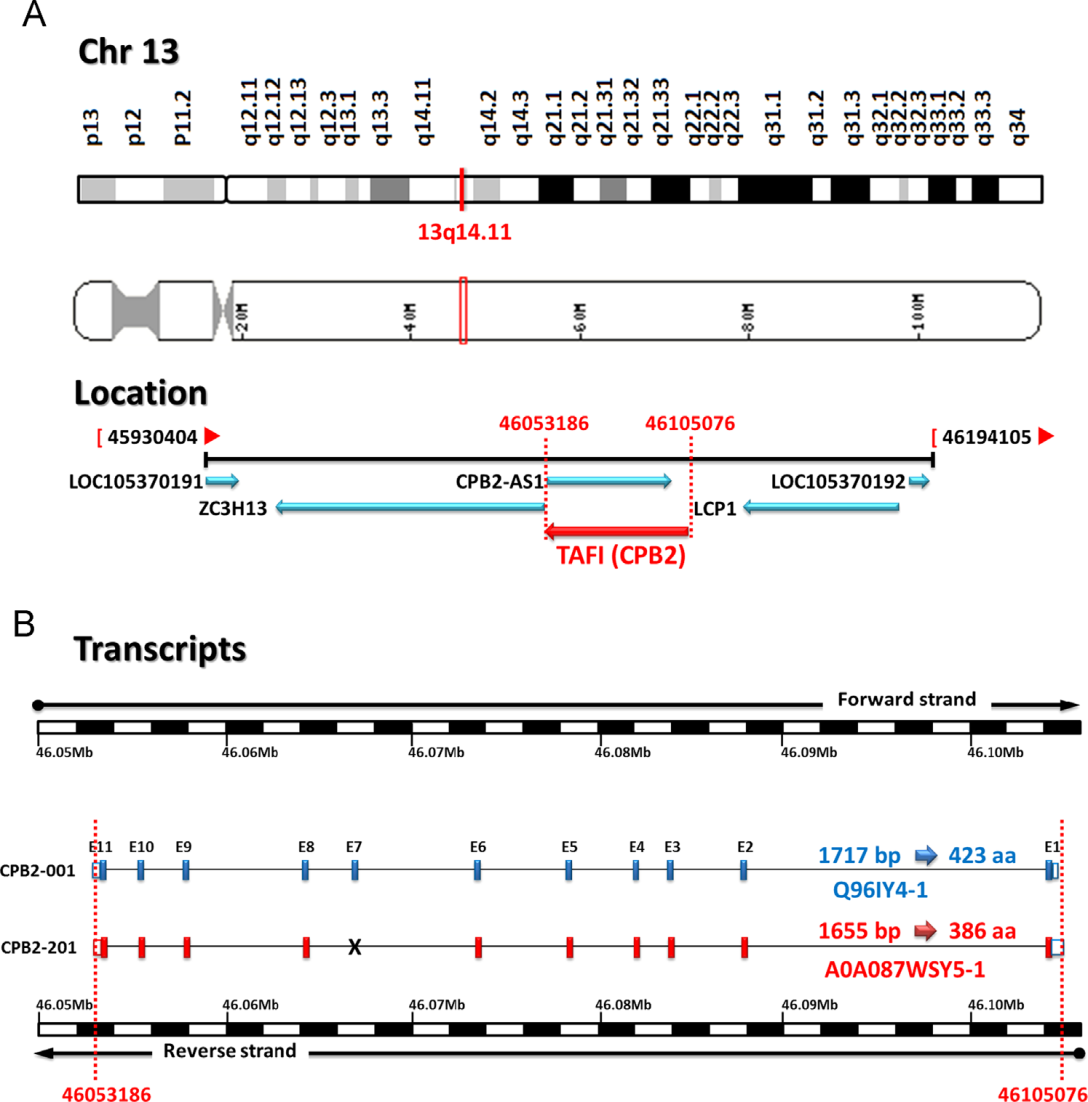
Genomic structure of the *TAFI* gene. (A) *TAFI* gene location. The *TAFI* gene (MIM 603101) is located in chromosome 13q14.11. The complete gene spans 58.190 kb of genomic DNA (NC_000013.11: Chr 13:46053186 to 46105076, complement strand; human genome assembly GRCh38.p2: Annotation release 107). LCP1, lymphocyte cytosolic protein 1 (L-plastin); LOC105370191 and LOC105370192, uncharacterized ncRNA; ZC3H13, zinc finger CCCH-type containing 13. (B) Splice variants of *TAFI* gene. The ENSG00000080618 gene (RefSeq gene ID 1361) has 2 transcripts due to alternative splicing. CPB2-001 transcript: Coding exons: 11, Transcript length: 1717 bp, Translation length: 423 residues (Q96IY4). CPB2-201 transcript: Coding exons: 10, Transcript length: 1655 bp, Translation length: 386 residues (A0A087WSY5-1). Transcript alignment between the two splice variants revealed extra 50 bases at 5′UTR, extra 1 base at 3′UTR, and lack of exon 7 of 111 bases in the CPB2-201 transcript (Data source: Ensembl.org and UniProtKB).

**Fig. 3:**
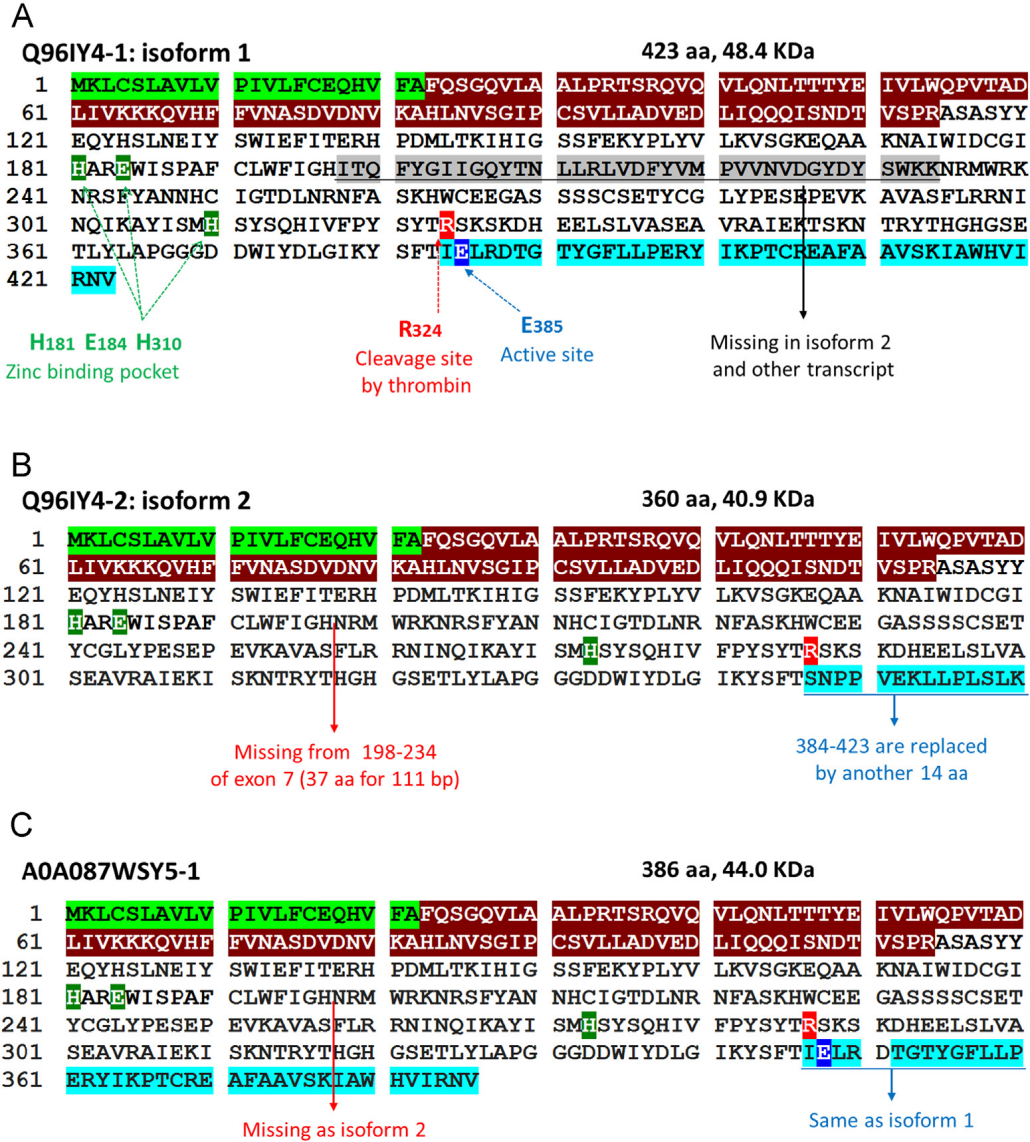
Amino acids sequence of TAFI protein isoforms. (A) Q96IY4-1 isoform. That is the main protein encoded by the gene; other forms are denoted as artifact (according to Vega Genome Browser release 62-Aug 2015 © WTSI / EBI and Consensus CDS protein set release 18). Letters highlighted green for signal peptide (22 residues: 1–22); maroon for activation peptide (92 residues: 23–114); clear color for main protein chain with catalytic activity (309 residues: 115–423). Zinc finger pocket (2 histidines and one glutamate), cleavage site by thrombin, and active site (catalytic residue: C-terminal glutamate) are indicated in the figure. (B) Q96IY4-2 isoform. Same sequence as isoform 1 except have missing 37 amino acids equivalent for exon 7 sequence from 198 to 234 residues and sequence distal to 384 till the end of isoform 1 is replaced by another 14 amino acids thus lacking the active site E385 essential for catalytic activity. (C) A0A087WSY5 protein share characteristics of Q96IY4-1 and Q96IY4-2. It has an identical sequence to isoform 1 except amino acids of exon 7 which are missing as in isoform 2. (Data source: UniProtKB for Q96IY4 and A0A087WSY5, last modified on July 22, 2015).

**Fig. 4:**
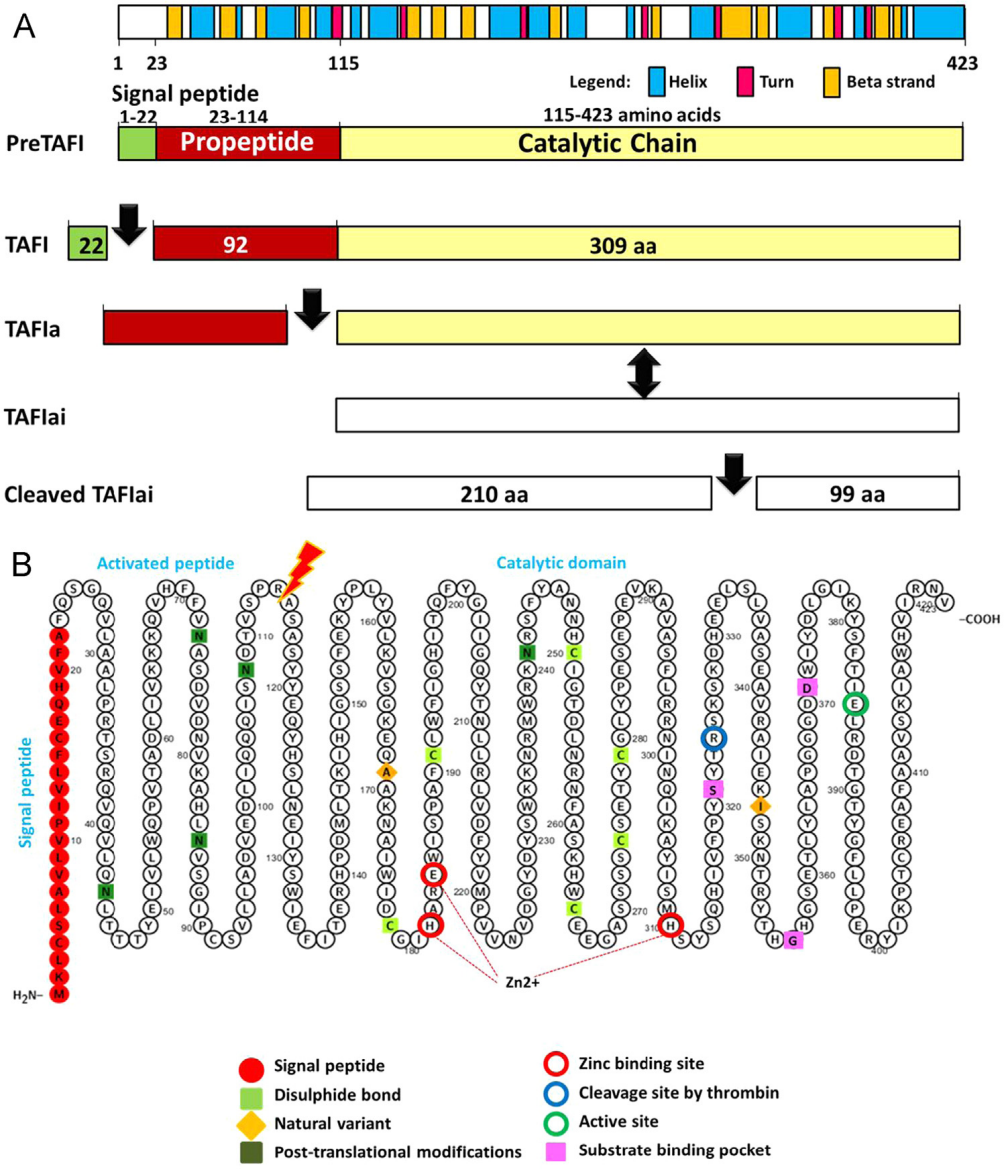
Secondary structure of TAFI protein. (A) Schematic representation of TAFI protein domains (UniProtKB Q96IY4-1). TAFI, Thrombin activatable fibrinolysis inhibitor; TAFIa, activated TAFI; TAFIai, inactive form. TAFI protein is a single chain glycoprotein zymogen of 423 amino acids long and molecular weight of 48.4 kDa that reach 60.0 kDa after glycosylation. It consists of three main domains; signal peptide (22 amino acids long), N-terminal activation peptide (92 amino acids), and a catalytic domain (309 amino acids), with a single molecule of zinc ion. Secondary structure features (helix, turn, and strand segments) are indicated (Data source: Ensembl and UniProtKB). (B) Graphical representation of the topology of TAFI generated with ProtterServer. It is a non-membranous protein secreted into the extracellular space. Red residues; 22 amino acids of the signal peptide. Green squares; N-glycosylation (PTMs; post-transcriptional modifications) 4 located at residues 44, 73, 85, and 108 of the activation peptide, and one in the catalytic domain at residue 241. Carbohydrates represent 19% of the protein components. Disulphide bonds between 178 and 191, 250 and 274, 265 and 279. The cofactor zinc coordination site (H181, E184, H310) and the basic C-terminal amino acid substrate binding pocket (S321, G358, D371) are indicated. Red arrow; site of proteolytic cleavage at Arg92–Ala93 bond by thrombin or plasmin, which results in the dissociation of the activated peptide (92 amino acids) from catalytic domain (309 amino acids) and exposure of the active site (Annotation data sources: UniProtKB, Phobius predictions, PeptideAtlas database) [12].

Thrombin-Activatable Fibrinolysis Inhibitor (TAFI) represents the molecular link between the coagulation and fibrinolytic pathways [4], [5] (Fig. 1). Human TAFI, also known as carboxypeptidase basic (CPB2) and unstable (CPU), is a procarboxypeptidase enzyme and a member of the family of metallocarboxypeptidases that carries a zinc ion essential for catalytic action and preferentially cleaves the carboxyl-terminal peptide bonds of basic amino acids [6], [7]. TAFI protein is encoded by TAFI gene at 13q14.11 spanning about 58 kb of genomic DNA. It has 2 different transcripts of 1717 and 1655 base pairs due to alternative splicing (Fig. 2). TAFI protein is synthesized by the liver as a single chain glycoprotein zymogen with a molecular weight of 60.0 kDa, circulates in the plasma in an inactive form bound to plasminogen [8], [9]. TAFI protein has 3 main domains; signal peptide of 22 residues, activation peptide (propeptide domain) of 92 amino acids, and catalytic chain of 309 residues (Fig. 3). Crystal structure of TAFI revealed that the precursor protein, in the zymogen form, exists as a globular domain followed by an extended alpha-helix. It maintains its stability via the interaction of the activation peptide with a highly dynamic region from residues 318–372 in the catalytic domain [10]. Dissociation of the activation peptide increases the dynamic flap mobility of this 55 residue segment and consequently results in increased plasticity of the entire catalytic chain, complete unfolding, and exposure of the cryptic thrombin-cleavage site present at Arg324 [11] (Fig. 4).

**Fig. 5:**
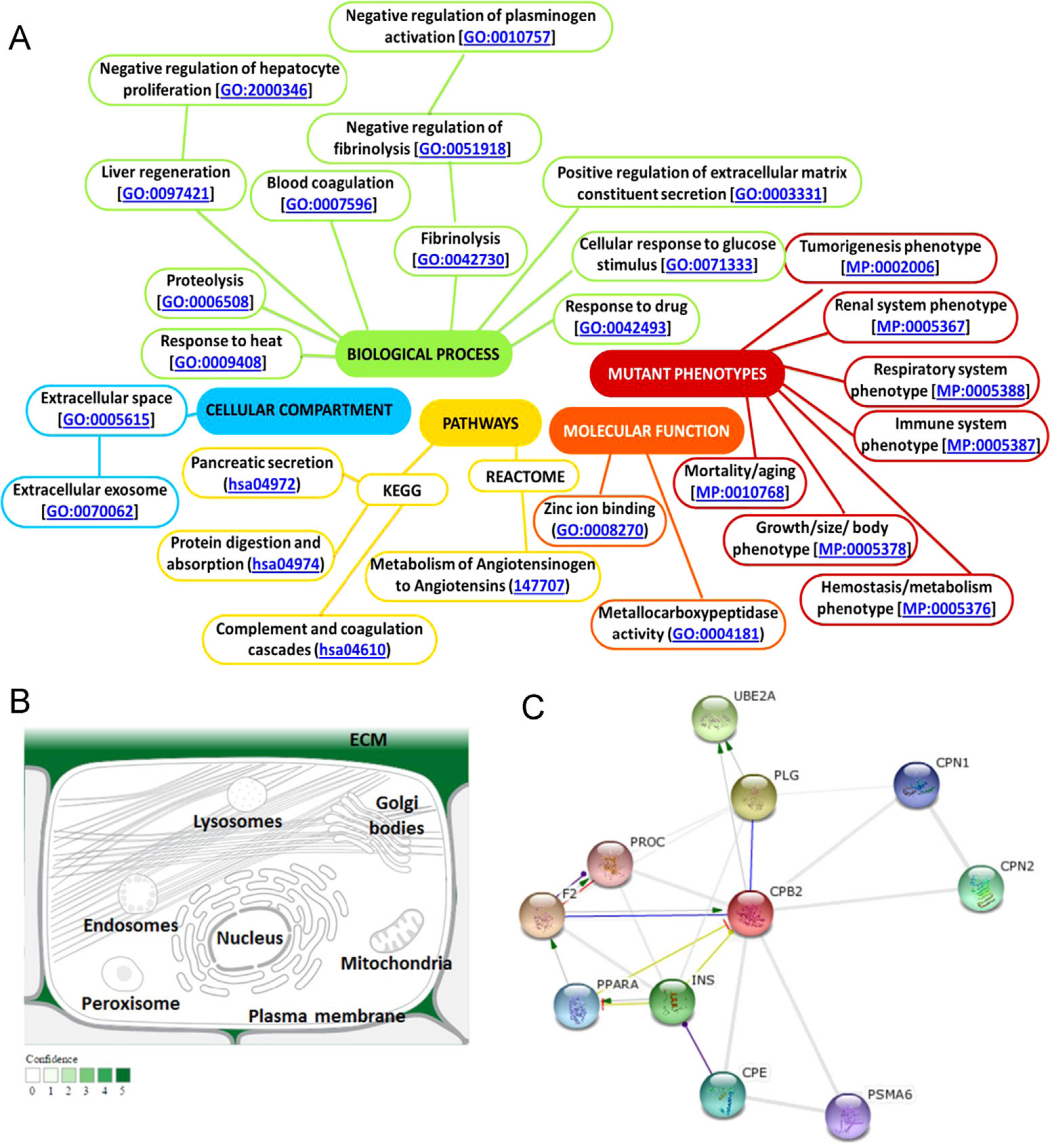
Functional activity of TAFI. (A) Gene ontology (GO) of human TAFI. The extracellular TAFI protein participates in multiple cellular processes [Data from: NextProt beta (http://www.nextprot.org) and GeneCards human gene database (http://www.genecards.org/) and MGI annotation for mammalian phenotype browser (http://www.informatics.jax.org/)]. (B) Subcellular localization of TAFI protein in the extracellular space. The confidence of the evidence is color coded, ranging from light green for low confidence to dark green for higher confidence. White indicates an absence of localization evidence (Data source: Compartment web server based on manually curated literature, high-throughput screens, automatic text mining, and sequence-based prediction methods: compartments.jensenlab.org/). (C) The STRING network view of TAFI protein. The network nodes are proteins. Predicted functional links between the proteins are indicated by edges. Modes of action are shown in different colored lines. A blue line indicates binding interaction; a grey line with green arrow shows proteins which activate TAFI protein; black edge states for reaction evidence. The string global score (confidence score) was adjusted to be greater than 0.95. CPE, carboxypeptidase E; CPN1, carboxypeptidase N, polypeptide 1; CPN2, carboxypeptidase N, polypeptide 2; F2, coagulation factor II (thrombin); INS, insulin; PLG, plasminogen; PPARA, peroxisome proliferator-activated receptor alpha; Ligand-activated transcription factor; PROC, protein C (inactivator of coagulation factors Va and VIIIa); PSMA6, proteasome (prosome, macropain) subunit, alpha type, 6; UBE2A, ubiquitin-conjugating enzyme E2A (http://string.embl.de/).

TAFI activity is generated during the blood clotting process via binding to thrombin, thrombin–thrombomodulin complex, or plasmin, which in turn cleave TAFI protein at Arg114 (residue Arg92 after removal of the signal peptide) into N-terminal activation peptide and catalytic domains, leading to exposure of the active site cleft of activated TAFI (TAFIa) [10], [13]. The rate of thrombin catalyzed activation of TAFI is increased by 1250 fold by formation of a ternary complex with thrombomodulin (T-TM-TAFI) rather than the binary thrombin–TAFI complex (T–TAFI) [14]. TAFIa exhibits carboxypeptidase activity by removal of the C-terminal lysine and arginine residues from fibrin that are essential for binding and activation of plasminogen. Consequently, removal of these residues leads to less plasmin formation and subsequently down-regulates fibrinolysis and stabilizing the clots [15]. TAFI is unique among carboxypeptidases in that the TAFIa decays spontaneously into the inactive form of TAFI (TAFIai) through a temperature-dependent conformational change, thus attaining a short half-life, a property that is crucial for its role in controlling blood clot lysis [10], [16]. In addition to the role of TAFI in fibrinolysis regulation, TAFIa also plays a role in the modulation of inflammation through down-regulation of pericellular plasminogen activation and inactivation of the inflammatory peptides bradykinin and anaphylatoxins C3a and C5a [16]. Other biological functions of TAFI were elucidated; including cell migration, blood pressure and tissue repair [4] (Fig. 5).

### 1.2 TAFI deregulation in cancer

The plasma concentration of TAFI appears to be under the control of genetic factors and non-genetic factors [16]. Several diseases, including diabetes, kidney transplantation, hypertension, nephritic syndrome, insulin resistance, obesity, and inflammatory bowel disease, have been shown to be positively associated with plasma TAFI levels [17]. In cancer, Expression Atlas databases reported over-expression of TAFI levels in breast, ovarian, and hepatic cancer cell lines [18], [19]. Plasma levels of TAFI were found to be significantly increased in various types of cancer; including BC [1], [20], lung cancer [21], gastric carcinoma [22], and multiple myeloma [23]. In addition, higher TAFI levels were associated with a more advanced cancer stage [1], [23]. The mechanism of increased circulating levels of TAFI in cancer patients remains to be fully explored. Two hypotheses were proposed; inflammatory cytokines induced by cancer cells may stimulate the production and secretion of TAFI from liver or vascular endothelial cells and thus increase its circulatory levels. Besides, malignant cells may also be a direct source of TAFI in cancer patients. Secretion of TAFI from cancer cells may increase intra-tumoral fibrin deposition and thus promotes tumor cell growth and dissemination [24].

### 1.3 Functional genetic variants of *TAFI* gene

**Fig. 6:**
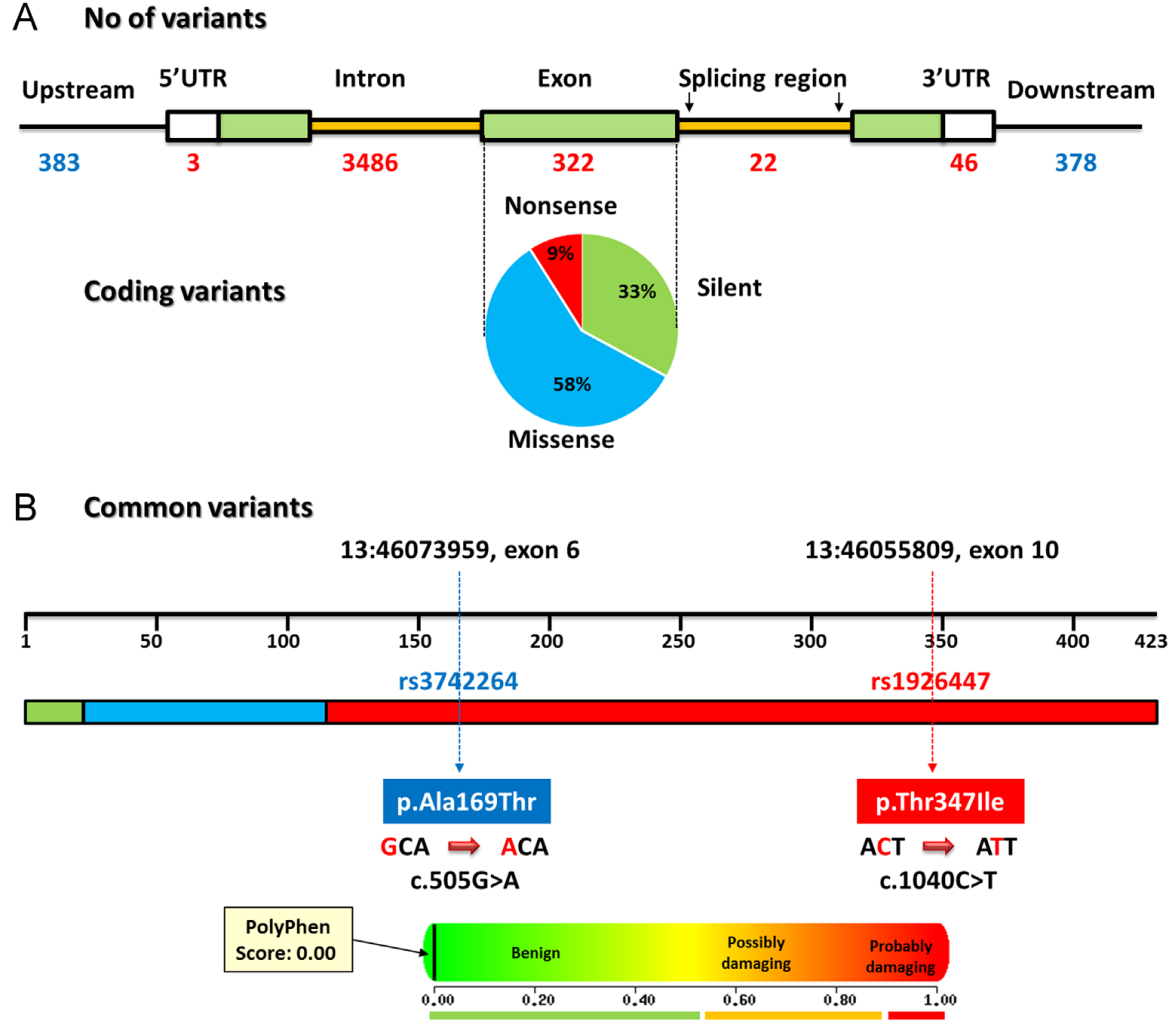
Genetic variants of *TAFI* gene. (A) Distribution of variants in *TAFI* gene. The types of coding polymorphisms are shown in the pie chart (Data source: human assembly GRCh38.p2 Annotation release 107; Ensembl.org). (B) Common variants in TAFI protein. *TAFI* gene contains 2 common variants rs3742264 and rs1926447 at positions 46073959 and 46055809 with MAF of 0.31 (T) and 0.22 (A), respectively. Predicted functional impact of A169T and T347I using PolyPhen server demonstrated them to be benign with a score of 0.00 (sensitivity: 1.0; specificity: 0.0) (Data source: dbSNP release 142).

According to several databases, TAFI gene contains multiple variants; all are rare with minor allele frequencies (MAF) of less than 0.001 except two common natural variants rs3742264 and rs1926447 at positions 46073959 and 46055809 with MAF of 0.31 (T) and 0.22 (A), respectively, Fig. 6. Although our *in silico* analysis predicted these two coding SNPs to be functionally neutral using polymorphism phenotyping (PolyPhen) version 2 and MutPred web-based programs [25], however, evidences from prior studies revealed that Thr169 and Thr347 are associated with higher plasma TAFI levels [5], [26] and that the 1040 T for C substitution results in a TAFIa species with a prolonged half-life at 37 °C [27]. Possibly, this increased stability of TAFIa compensates for the decreased levels of the protein [5].
